# Evolutionary diversification of the BetaM interactome acquired through co-option of the ATP1B4 gene in placental mammals

**DOI:** 10.1038/srep22395

**Published:** 2016-03-04

**Authors:** Tatyana V. Korneenko, Nikolay B. Pestov, Nisar Ahmad, Irina A. Okkelman, Ruslan I. Dmitriev, Mikhail I. Shakhparonov, Nikolai N. Modyanov

**Affiliations:** 1 Shemyakin and Ovchinnikov Institute of Bioorganic Chemistry, Moscow 117871, Russia; 2 Department of Physiology and Pharmacology and Center for Diabetes and Endocrine Research, University of Toledo College of Medicine, 3000 Arlington Ave, Toledo, OH 43614, USA

## Abstract

ATP1B4 genes represent a rare instance of orthologous vertebrate gene co-option that radically changed properties of the encoded BetaM proteins, which function as Na,K-ATPase subunits in lower vertebrates and birds. Eutherian BetaM has lost its ancestral function and became a muscle-specific resident of the inner nuclear membrane. Our earlier work implicated BetaM in regulation of gene expression through direct interaction with the transcriptional co-regulator SKIP. To gain insight into evolution of BetaM interactome we performed expanded screening of eutherian and avian cDNA libraries using yeast-two-hybrid and split-ubiquitin systems. The inventory of identified BetaM interactors includes lamina-associated protein LAP-1, myocyte nuclear envelope protein Syne1, BetaM itself, heme oxidases HMOX1 and HMOX2; transcription factor LZIP/CREB3, ERGIC3, PHF3, reticulocalbin-3, and β-sarcoglycan. No new interactions were found for chicken BetaM and human Na,K-ATPase β1, β2 and β3 isoforms, indicating the uniqueness of eutherian BetaM interactome. Analysis of truncated forms of BetaM indicates that residues 72-98 adjacent to the membrane in nucleoplasmic domain are important for the interaction with SKIP. These findings demonstrate that evolutionary alterations in structural and functional properties of eutherian BetaM proteins are associated with the increase in its interactome complexity.

Molecular evolution of ATP1B4 genes is a rare instance of orthologous gene co-option^1^ creating fundamental changes in properties of the encoded BetaM proteins. These proteins in fishes, amphibians and birds are authentic Na,K-ATPase β-subunits which in complex with α-subunits function as ion pumps in the plasma membrane^2^. In placental mammals, BetaM proteins retain all the structural features and signature motifs of X,K-ATPase β-subunits, which are type II membrane glycoproteins, but acquire entirely different, long (over 100 amino acid residues) N-terminal domains containing two extended Glu-rich clusters and a N-terminal Arg-rich nonapeptide^2^,^3^,^4^,^5^,^6^. Eutherian BetaM lost the ability to assemble with an α-subunit into the Na,K-ATPase molecule and became a resident of the inner nuclear membrane exposing the evolutionary acquired N-terminal domain to the nucleoplasm^2^,^5^,^6^,^7^,^8^. The Arg-rich N-terminus of the nucleoplasmic domain most probably is a nuclear localization signal^9^. In contrast to other members of Na,K-ATPase β-subunit family, the polypeptide chain of eutherian BetaM is highly sensitive to degradation by endogenous proteases. Short high-mannose type N-linked oligosaccharides are a characteristic feature for short-lived proteins^10^,^11^. Glu-rich clusters are typical homopolymeric amino acid repeats that usually form intrinsically disordered domains serving as flexible molecular recognition elements in many signaling proteins and transcription regulators that are capable to interact with various binding partners for diverse cellular functions^12^,^13^.

Expression of BetaM is confined to skeletal and cardiac muscle, and is strictly developmentally regulated, being the highest in perinatal skeletal myocytes, but is practically undetectable in muscles of adult mice. This specific ontogenic regulation, especially the transient pattern of expression, indicates that BetaM functions are highly specialized and related exclusively to concurrent developmental events.

We demonstrated that neonatal skeletal muscle BetaM associates with nuclear transcriptional co-regulator SKIP. Through interaction with SKIP, BetaM is involved in regulation of gene expression as exemplified by increase of expression of inhibitory Smad7, implicating BetaM in regulation of the TGF- β signaling pathway^2^. All these unique characteristics acquired through ATP1B4 gene co-option indicate that BetaM represents a novel eutherian-specific transcriptional co-regulator that plays an essential role during the critical period of perinatal development.

To elucidate evolution of the BetaM interaction network we performed an expanded analysis of BetaM interactome. The protein interactions of eutherian BetaM demonstrate that alterations in its structural and functional properties results in a sharp increase of interactome complexity. These findings provide new insights for further characterization of the BetaM functions in placental mammal development and a better understanding of the necessity and physiological importance ATP1B4 gene co-option.

## Results

To map binary protein interactions of BetaM, we have rescreened mouse embryo cDNA libraries in a yeast two-hybrid system with full-length BetaM. Seventeen proteins were positive in the first screen. In the second round, proteins were classified into three groups according to our criteria of confidence (Supp. Table 1, Supp. Fig. 1). High-confidence clones were those that demonstrated fast growth on High Stringency medium and positive reaction for α-galactosidase and met none of the criteria for lowering confidence in subsequent tests. Medium-confidence clones showed insufficient specificity to BetaM, e.g. have noticeable growth with other X,K-ATPase β-subunits, if the identified prey is only a small fragment of the full length protein, and if later homologues of the prey gave clear false-positive reactions. The low-confidence group included those that consistently grew only on a Low Stringency medium (SD/-3) medium. Incorrect topology of transmembrane segments (either determined or predicted) with respect to the connection site with the GAL4 DNA binding domain, which should be located inside the nucleus, was a basis to classify such clones as low-confidence. Interestingly, BetaM itself was identified as an interactor. This fact is sufficient to claim that yeast two-hybrid screening using proteins with one transmembrane segment is capable to identify true interactors. Also, this indicates that BetaM exists in vivo as a dimer or oligomer. We also screened a human library using split ubiquitin system, which is sometimes regarded as better fit for membrane proteins (Supp. Table 2). Only β-sarcoglycan represented the overlap between the results of the two experiments. This result is not surprising since the conventional yeast two-hybrid requires the interaction to occur inside the nucleus, whereas the split-ubiquitin system relies on cytoplasmic events. The interaction with β-sarcoglycan should be labeled as the highest confidence since it gave positive results in two screenings using independent approaches. All results have been depicted in Table 1.

Screening of the same library with mouse β1, β2 and β3 using the same procedure as with BetaM yielded no interactors (results not shown). This indicates that BetaM is unique among its structural family in its ability to interact with most of the identified proteins. Also, other β-subunit isoforms seem to have simple interactome profiles – this is easily explainable by the consideration that they evolved to specifically interact with the Na,K-ATPase and H,K-ATPase α-subunits.

We were interested if the BetaM interactome was conserved in non-mammalian animals. We screened a whole chicken embryo cDNA library with chicken BetaM but found no positive interactors. Because this may indicate that exactly the same clones as in case of mammals are absent from the chicken library, we cloned chicken homologs of all high-confidence and some of the medium confidence BetaM interactors. These were tested cross-wise together with chicken and mouse BetaM and the mouse BetaM interactors. Results (Supp. Table 3) indicate that, except for chicken β-sarcoglycan, none of them interact as assigned by our criteria for the library screening. The result suggests that among the high-confidence interactions (Supp. Table 1) only the BetaM – β-sarcoglycan pair is conserved among mammals and birds. Previously described interaction BetaM - SKIP seems to be absent in the chicken.

**Figure 1:**
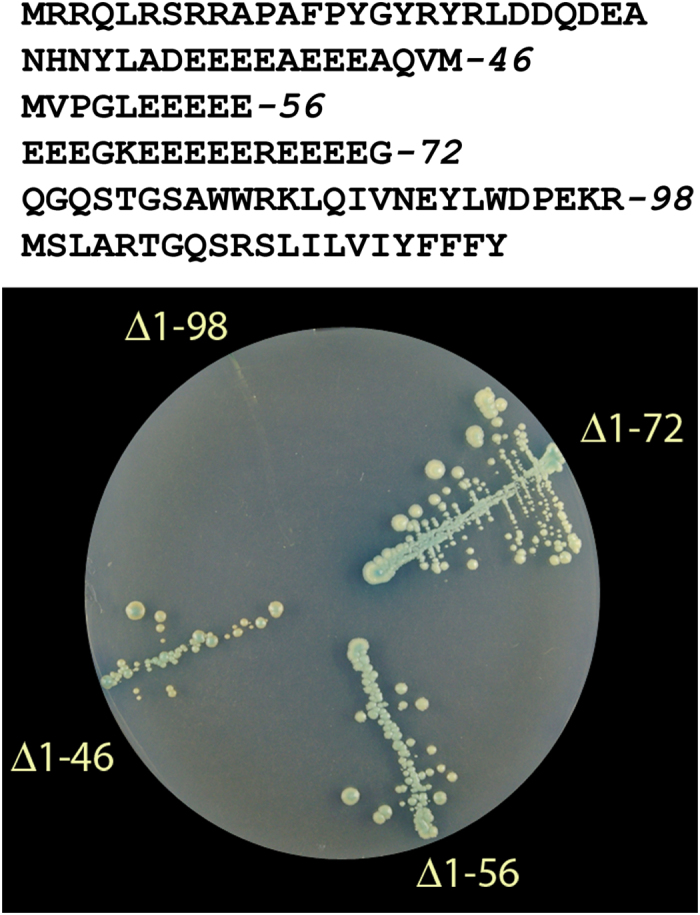
Deletion mapping of SKIP interaction site. The amino acid sequence of BetaM protein shown on top with coordinates of the truncations. Lower panel – results of two-hybrid test of the BetaM truncation mutants in pair with SKIP. Negative control experiments with yeast harboring plasmids encoding SKIP mutant and an empty vector yielded no cell growth (not shown).

N-terminal truncation of 56 residues, which removes the N-terminal Arg-rich peptide and a half of Glu-rich stretch, does not abolish the dimerization of BetaM. The truncation does not disrupt any of the identified interactions. Transmembrane and/or C-terminal domains are required for interactions with LZIP, Syne, reticulocalbin and β-sarcoglycan; and N-terminal domain is sufficient for interaction with SKIP, LAP-1, PHF-3, heme oxidases and may participate in other interactions. Deletion mutants of the N-terminal domain of BetaM showed that removal of either N-terminal arginine rich stretch or also of the two GARs (glutamic-acid rich regions) cannot abolish interaction with SKIP (Fig. 1). A fragment 72–98 between the GARs and the transmembrane domain (Fig. 1) is required for the interaction with SKIP. One cannot exclude the possibility that an increase in affinity of interaction with SKIP is conferred by the GARs. It is interesting, that this region is composed of two portions: one highly variable and another quite conserved (Suppl. Fig. 2)

LAP1 and BetaM predominantly co-localize in protrusions of the nuclear membrane in mouse myoblast cells (Suppl. Fig. 3). This example illustrates that the proteins classified as medium confidence interactors may indeed show interaction under conditions different from yeast two-hybrid experimental settings.

## Discussion

Evolution of interactomes is an intensively studied area where many gaps need to be filled. In particular, the interactomes of membrane proteins have been poorly studied. The ATP1B4 gene and the BetaM protein constitute an interesting example in many respects. Considering peculiarities of the interactome (interaction with SKIP and lack of association with Na,K-ATPase) and tissue-specific expression of BetaM, especially its highly specifc spaciotemporial expression in mammals, we decided to address the question of how evolutionary changes in tissue-specific expression and interactome may be correlated in the case of BetaM protein.

**Figure 2:**
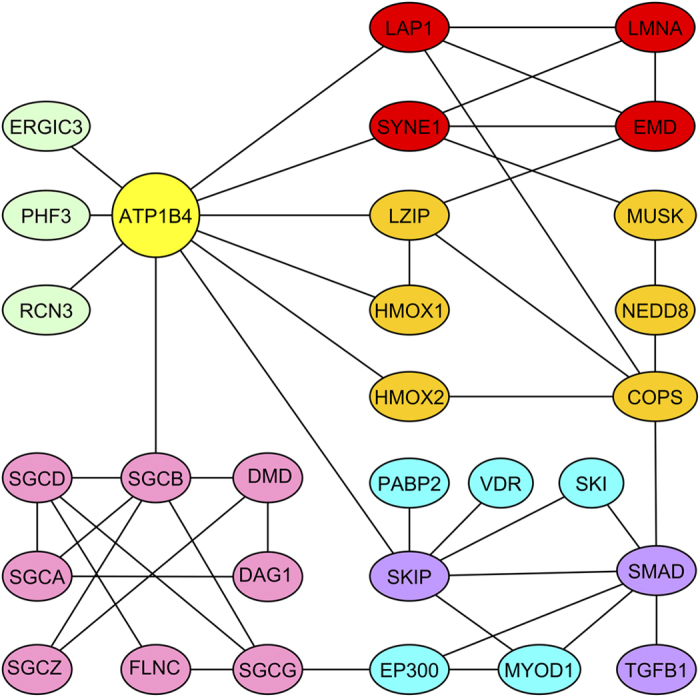
Interactome of the mammalian BetaM. The binary interactions shown here are explicated in Suppl. Table 4. This scheme is focused on the interconnectivity of different nodes of the BetaM interactome, with attention to two tissues: skeletal muscle and heart. Some of them have many more putative interactors, for example, SKIP may bind about one hundred different proteins. Components of the nuclear membranes marked red, members of the dystrophin-associated glycoprotein complex - pink, participants of the previously demonstrated link between ATP1B1 and TGFβ – magenta, some SKIP-interacting proteins of importance to skeletal muscle – cyan, proteins with little known functions – light-green, various other proteins – dark-yellow, ATP1B4 - gold.

We compile the data with known interactions to build an extended BetaM interactome map (Fig. 2.). Elongation of the N-terminal domain of BetaM during mammalian evolution cannot be considered the sole mechanism for the expansion of the number of interactors of BetaM. For example, presence of all domains in the full-length BetaM is required for the interaction with β-sarcoglycan (SGCB).

Integrating interactome and tissue-specificity is now widely recognized as important for the understanding of normal development and pathogenesis^14^. The seminal bioinformatic paper on the evolution of tissue-specific interactome posits that evolutionary narrowing of the tissue-expression profile of a gene is usually associated with decreasing the number of protein-protein interactions for the encoded protein^15^. Here, we found that BetaM provides an interesting case of expansion of the interactome synchronously with a strict specialization of its spatiotemporial expression. Indeed, only β-sarcoglycan is a BetaM interactor in the chicken. It may be concluded that specialization of the tissue-specific expression (muscle, brain and other tissues in the chicken , and only striated muscle in mammals) coincided with the acquisition of new interactions; in other words, increases in affinity of previously weak ones.

Many of the identified high and medium confidence interactors play important roles in muscle pathologies. For example, limb-girdle muscular dystrophies (LGMDs) represent a clinically and genetically heterogeneous group of diseases which are characterized by progressive weakness of the pelvic and shoulder girdle muscles. LGMD2E mutation (β-sarcoglycan mutation) was identified in an Amish family^16^. SGCB-/- deficient mice exhibited decreased muscle specific force and calcium transients, and displayed reduced exercise capacity^17^. The SGCB-null mouse, with knocked-down β-sarcoglycan, develops severe muscular dystrophy as in type 2E human limb girdle muscular dystrophy with fibrosis^18^. Mice lacking the KASH domain of Syne-1 display a myopathic phenotype similar to that observed in human patients^19^. Surviving mice suffer from hind limb weakness and an abnormal gait. With increasing age, kyphoscoliosis, muscle pathology and cardiac conduction defects develop^20^. Striated muscle-selective depletion of lamina-associated polypeptide 1 (LAP1), an integral inner nuclear membrane protein, leads to profound muscular dystrophy with premature death in mice as well as left ventricular systolic dysfunction^21^,^22^.

With respect to functions of SKIP specific to skeletal muscle^23^, it is interesting that poly(A)-binding protein 2 (PABP2, oculopharyngeal muscular dystrophy gene) interacts with SKIP and stimulates muscle-specific gene expression. PABP2 co-operated with SKIP to synergistically activate E-box-mediated transcription through MYOD, a key myocyte differentiation player^24^.

The effect of BetaM on TGFβ signaling through interaction with SKIP, the role for which BetaM has been already established^2^. BetaM may also act through other pathways involved in skeletal and cardiac muscle pathologies. Recently, a link between a sarcoglycanopathy in muscles and excessive TGFβ signaling was described that is mediated through SMAD2-3^25^. Moreover, inhibition of TGFβ signaling protects skeletal muscle from injury and dystrophic disease in δ-sarcoglycan knocked out mice^26^.

BetaM appears to link sarcolemmal and nuclear constituents that are important for structural integrity and signal transduction in muscle cells. It is of interest that sarcoglycans, although predominantly sarcolemmal proteins, can be found in the nuclear envelope^27^. Moreover, they can accumulate in the nucleus in certain conditions; for example, retention of mutant δ-sarcoglycan in the nucleus is accompanied by partial nuclear sequestration of β- and γ-sarcoglycans^28^. Some of the extranuclear proteins may undergo translocation into the nucleus upon certain stimuli. For example, LZIP contains a basic leucine zipper domain that mediates DNA binding. LZIP is primarily associated with the ER membrane but, in response to ER stress or cell injury, the N-terminus of LZIP is liberated from the ER by proteolytic cleavage and translocates to the nucleus, leading to the activation of its target genes^29^,^30^.

Many of these interactors have links to the COPS5 component of protein-degrading signalosome. This may indicate that the BetaM protein, which is very sensitive to endogenous proteolysis^10^, may be targeted to the signalosome through one of these interactions.

Phylogenetically, BetaM belongs to X,K-ATPase β-subunit family. This family includes Na,K-ATPase, gastric H,K-ATPase and nongastric H,K-ATPase that contain a catalytic α subunit, and the β-subunit plays an accessory role. Major functions such as ATP binding, phosphorylation and ion transport are performed by α-subunit, whereas the α-β interaction is required for maturation, stability and translocation of the ATPases to the plasma membrane, potassium affinity and cardiac glycoside sensitivity^3^,^31^,^32^,^33^,^34^. The lack of association of mammalian BetaM with a Na,K-ATPase α-subunit created an interesting dilemma from ontogenetic point of view: has BetaM lost this ability during evolution and speciated from other subunit isoforms or the α-β association was not an essential feature of all isoforms? This issue may have implications for understanding of the multiplicity of functions of Na,K-ATPase subunit isoforms. Other functions outside binding the α-subunit cannot be excluded for other β-subunits, but so far BetaM seems to be unique.

In summary, identification of BetaM partners provides a basis for further characterization of the BetaM functions in the development of placental mammals and better understanding of the physiological importance ATP1B4 gene co-option.

## Materials and Methods

### DNA cloning

Truncation mutations were made by inverse PCR with a high fidelity DNA polymerase (Phusion, New England Biolabs, USA) supplemented with *Dpn* I treatment to digest the template plasmid, ligation and transformation. Yeast transformation has been achieved by standard lithium acetate – PEG method.

### Yeast Two-Hybrid Screening

Pretransformed BD Matchmaker 17 day mouse embryo cDNA library in yeast Y187 cells (BD Biosciences) has been screened according to manufacturer’s protocol. To construct the bait plasmid, full-length mouse BetaM was amplified (using primers Forward: TGAACACATATGAGACGGCAACTCCGCT, Reverse: TTCCTAGGTTTCTATGTTCAG) and cloned in-frame with the GAL4 DNA binding domain of pGBKT7 vector at *Nde* I/*Sma* I sites. The bait-transformed yeast AH109 cells were mated with Y187 cells containing cDNA library and were grown on SD/-Trp/-Leu/-His plates for 5 days at 30 °C. Primary colonies were regrown several times on high stringency selection medium (SD/-Ade/-Trp/-Leu/-His) with α-X-Gal to select for induction of reporter genes (His, ADE and MEL1) and then passed several times on SD/-Leu/-Trp plates with a-X-Gal. The identified clones were considered positive if they showed fast growth on a selective medium as well as positive reaction for α-galactosidase on both selective or unselective media with no observable growth in combination with empty bait plasmid. The interaction of the hybrid proteins was examined by co-transformation of the prey and bait plasmids into AH109 followed by the selections on SD/-Ade/-Trp/-Leu/-His with X-a-Gal plates and further confirmed by mating of AH109 and Y187 cells transformed with bait or isolated prey plasmids, respectively. The pGBKT7 “empty” vector was used as a negative control. By doing so, the colonies obtained were tested to exclude obvious false positives using retransformation with bait and control plasmids into fresh yeast cells and the test repeated with or without bait. Plasmids from positive clones were rescued into *E. coli* and sequenced.

### Yeast Split-Ubiquitin Screening

Full length ORF of human BetaM has been cloned into pBT3 vector at its two *Sfi I* sites. A human lung library was screened using a split-ubiquitin system (Mobitech, Switzerland) according to manufacturer’s instructions and overall strategy similar to the procedures for the standard two-hybrid system.

### Co-localization of tagged proteins in living cells Cell culture and transfection by m-green fluorescent protein

The plasmid that produces splice variant A of BetaM C-terminally fused with enhanced green fluorescent protein (GFP) was described before^6^. Plasmid encoding LAP1 C-terminally fused red fluorescent protein was constructed in a similar way. C2C12 mouse myoblasts were grown in glass-bottomed chambers and transfected with the plasmids in complex with Superfect (Qiagen, Valencia, CA) and incubated for 8 h. Images were collected with a DMIRE2 laser scanning microscope (Leica, Mannheim, Germany).

## Additional Information

**How to cite this article**: Korneenko, T. V. *et al*. Evolutionary diversification of the BetaM interactome acquired through co-option of the ATP1B4 gene in placental mammals. *Sci. Rep.* **6**, 22395; doi: 10.1038/srep22395 (2016).

## Supplementary Material

Supplementary Information

## Figures and Tables

**Table 1:** BetaM interactors identified by screening cDNA libraries

**Interactor**	Amino acidcoordinates	**Domains**	Tissue-specificexpression	Comments on known functions,especially in striated muscles
BetaM/ATP1B4	57–352	N-term. truncated	Striated muscle	Putative di- oligomerization of the BetaM protein
SKIP/SNW1	1–330	Full length	Ubiquitous	Serves as a docking site for both transcription co-activators and co-repressors, modulate transcription elongation and splicing^23^
Sarcoglycan β/SGCB	1–330	Full length	High in skeletal muscle, heart	Component of the dystrophin-associated glycoprotein complex (DAGC), a mechanosignaling complex abundant in sarcolemma^35^
LZIP/CREB3	226–402	C-terminal excluding basic leucine zipper	Ubiquitous	Activates transcription from the unfolded protein response element by proteolytic cleavage in the ER translocation to the nucleus^28^,^29^
Syne-1/nesprin-1	566–949	KASH domain containing	Ubiquitous, high in skeletal muscle	Transmembrane protein of the inner nuclear membrane, anchors nuclei at the neuromuscular junction, facilitates nuclear movements^36^
Reticulocalbin 3/RCN3	1–328	Full length	Ubiquitous	Ca-binding luminal protein of the ER^37^
LAP1/TOR1AIP2	96–520	N-term. truncated	Ubiquitous, high in placenta	Transmembrane protein of the inner nuclear membrane^38^
Heme oxidase 1/HMOX1	200–389	N-term. truncated	Ubiquitous, high in heart	Cytoprotective, anti-oxidant enzyme degrading heme to biliverdin, inhibits differentiation and increases viability of skeletal myoblasts^39^
Heme oxidase 2/HMOX2	1–315	Full length	Ubiquitous	May be similar to HMOX1
PHF3	168–397	N-term. truncated	Ubiquitous, high in heart	Zinc-finger protein with unknown function^40^
ERGIC3	1–383	Full length	Ubiquitous, high in skeletal muscle	Endoplasmic reticulum-Golgi intermediate compartment protein, enhances ER-stress resistance^41^
