# Supplementary material for: Evolutionary diversification of the BetaM interactome acquired through co-option of the ATP1B4 gene in placental mammals
Source: Sci Rep. 2016 Mar 4;6:22395. doi: 10.1038/srep22395 (PMC4778017; doi:10.1038/srep22395)
Supplement: Supplementary Information [file srep22395-s1.doc]

**SUPPLEMENTARY INFORMATION**

**Evolutionary Diversification of the BetaM Interactome**

**Acquired through Co-option of the ATP1B4 Gene in Placental Mammals**

Tatyana V. Korneenko^1,2^, Nikolay B. Pestov^1,2^***, ,Nisar Ahmad^2^, Irina A. Okkelman^1^, Ruslan I. Dmitriev^1^, Mikhail I. Shakhparonov^1^, Nikolai N. Modyanov^2^,***

^1^ *Shemyakin and Ovchinnikov Institute of Bioorganic Chemistry, Moscow 117871, Russia*

*^2^* Department of Physiology and Pharmacology and *Center for Diabetes and Endocrine Research,* University of Toledo College of Medicine, 3000 Arlington Ave, Toledo, OH 43614, USA.

***Corresponding authors. Fax:+7-495-330-6556 (N.B. Pestov), +1-419-383-2871 (N.N. Modyanov).

E-mail addresses: korn@mail.ibch.ru (N.B. Pestov), nikolai.modyanov@utoledo.edu (N.N. Modyanov).

**SUPPLEMENTAL TABLE 1**

**BetaM interactors identified by conventional yeast two-hybrid screening a mouse embryo cDNA library**

| N | Identity | Amino  acid  coordinates | **Test1** | | **Test 2** | | | | | | | | |
| --- | --- | --- | --- | --- | --- | --- | --- | --- | --- | --- | --- | --- | --- |
|  |  |  | no bait | βm | βm full | βm N | βm C | β1 | β2 | β3 | swap | βm △1-56 | Criteria |
| High confidence | | | | | | | | | | | | | |
| 1 | **βm** (AF348325) | 52-352C  57-352C | 🞇  🞇 | ●  ● | ●  ND | ND  ND | ND  ND | 🞇  ND | 🞇  ND | 🞇  ND | ●  ND | ●  ND |  |
| 2 | **SKIP, SNW1** (NM_025507) | N-330C | 🞇 | ● | ● | ● | 🞇 | 🞇 | 🞇 | 🞇 | ND | ● |  |
| 3 | **Syne1, Nesprin-1** (NM_022027) | 479-949C  566-949C | 🞇  🞇 | ●  ● | ●  ND | ◗  ND | 🞇  ND | 🞇  ND | 🞇  ND | 🞇  ND | ●  ND | ●  ND |  |
| 4 | Sarcoglycan β, SGCB (P82349) | N-320C | 🞇 | ● | ● | 🞇 | 🞇 | 🞇 | 🞇 | 🞇 | ● | ● |  |
| Medium confidence | | | | | | | | | | | | | |
| 5 | **LZIP, СREB3, Luman** (NM_013497) | 226-402C  143-402C | 🞇  🞇 | ●● | ●  ND | 🞇  ND | 🞇  ND | 🞇  ND | 🞇  ND | 🞇  ND | 🞇  ND | ●  ND | 1, 2 |
| 6 | **Reticulocalbin 3, RCN3**  (NM_026555) | N-328C | 🞇 | ● | ● | 🞇 | 🞇 | 🞇 | 🞇 | 🞇 | ● | ● | 1 |
| 7 | **LAP1, Tor1aip2** (BC010841) | 96-520C | 🞇 | ● | ● | ● | 🞇 | 🞇 | ◗ | ◗ | ● | ◗ | 3 |
| 8 | **Heme oxidase 1, HMOX1** (BC010757) | 161-289C  200-289C | 🞇  🞇 | ●  ● | ●  ND | ●  ND | ◗  ND | 🞇  ND | ◗  ND | 🞇  ND | ◗  ND | ●  ND | 3 |
| 9 | **Heme oxidase 2, HMOX2** (AAC17981) | N-315C | 🞇 | ● | ● | ● | 🞇 | 🞇 | ● | 🞇 | ND | ND | 3 |
| 10 | **PHD finger , PHF3** (XM_129836) | 168-397 | 🞇 | ● | ● | ● | 🞇 | 🞇 | ● | 🞇 | ND | ND | 3, 4 |
| Low confidence | | | | | | | | | | | | | |
| 11 | **Cadherin 16, CDH16** (NM_007663) | 266-430C | 🞇 | ● | ● | ND | ND | 🞇 | ● | 🞇 | ND | ● | 3,6 |
| 12 | **Frizzled 1, FZD1** (O70421) | 288-642C | 🞇 | ◗ | ND | ND | ND | ND | ND | ND | ND | ND | 5,6 |
| 13 | **Unnamed (**BAC35045) | 90-282C | 🞇 | ● | ● | ● | 🞇 | ND | ND | ND | ND | ND | 6 |
| 14 | **SC65, LEPREL4** (BC031856) | 242-436C | 🞇 | ◗ | ND | ND | ND | ND | ND | ND | ND | ND | 5 |
| 15 | **Tetraspanin 3, TSPAN3** (AF133427) | 160-253C  107-253C | 🞇  🞇 | ◗  ● | ND  ND | ND  ND | ND  ND | ND  ND | ND  ND | ND  ND | ND  ND | ND  ND | 5,6 |
| 16 | **TGN29** (AAH34829) | 109-251C | 🞇 | ◗ | ND | ND | ND | ND | ND | ND | ND | ND | 5 |
| 17 | **Tmp21-I, TMED10** (O35587) | 39-219C | 🞇 | ● | ND | ND | ND | ND | ND | ND | ND | ND | 6 |

**Test 1** describes behaviour of the clones in the cDNA library screening as detailed in Methods. ● ‑ growth on High Stringency medium (- 4), ◗ ‑ positive only on Low Stringency medium (- 3), 🞇 ‑ negative. All clones gave also positive α-galactosidase reaction on nonselective medium indicating activation of the promoter even without selection pressure. **Test 2** represents reassaying on High Stringency medium with different bait proteins and BetaM (βm) fragments. BetaM N and C indicate N-terminal cytodomain and C-terminal ectodomain, respectively. Swap – test with reversed bait and prey fusions. ND – not determined. **Criteria** for decreasing confidence: 1 – false-positive homologues, 2 – poor growth in swap assay, 3 – low specificity, 4 – small fragment of a large protein, 5 – positive only on low stringency medium, 6 – membrane topology problems.

**SUPPLEMENTAL TABLE 2**

**Putative BetaM interactors identified by split ubiquitin screening a human cDNA library**

| N | Identity | Amino  acid  coordinates | Criteria |
| --- | --- | --- | --- |
| **High confidence** | | | |
| 1 | Sarcoglycan β, SCG-β, SGCB, (NP_000223) | N-318C |  |
| **Medium confedence** | | | |
| 2 | **ERGIC3, Endoplasmic Reticulum-Golgi Intermediate Compartment Protein, breast cancer antigen 84** (NP_057050) | N-383C | 1 |
| **Low confidence** | | | |
| 3 | **B-cell receptor-associated protein 31 isoform a, BCAP31** (NP_001132929) | 26-313C | 1,2 |
| 4 | **Uncharacterized protein C4orf3 isoform 1** (NP_001163801) | 131-199C | 1,2 |
| 5 | **Surfactant C, SFTPC** (EAW63705) | 39-240C | 1,3 |
| 6 | **genome fragment** | IIILFITFHSYQLLFLLFASFYILWFYSLLFFRVSR | 1,3 |
| 7 | **Tetraspanin CD63, TSPAN30, melanoma antigen** (NP_001771) | N-238C | 1,3 |

**Criteria** for decreasing confidence: 1 – lack of other support, 2 ‑ small fragment of a large protein, 2 – not expressed in muscle, 3 – membrane topology problems.

**SUPPLEMENTAL TABLE 3**

**Analysis of interaction between mouse and chicken βm and SKIP**

|  | Mouse | | | | Chicken | Control |
| --- | --- | --- | --- | --- | --- | --- |
| GAD  GBK | βm | βmΔ1-56 | SKIP | Syne-1 | βm |
| Mouse βm | ● | ● | ● | ● | ● | 🞇 |
| Mouse βmΔ1-56 | ● | ● | ● | ● | ND | 🞇 |
| Mouse Syne-1 | ● | ● | ND | ND | ND | 🞇 |
| Mouse SCG-β | ● | ● | ND | ND | ● | 🞇 |
| Chicken βm | ND | 🞇 | ◗ | ◗ | ◗ | 🞇 |
| Chicken SKIP | 🞇 | 🞇 | 🞇 | ND | 🞇 | 🞇 |
| Chicken sarcoglycan-β | ND | ● | ND | ND | ● | 🞇 |

● ‑ growth on High Stringency medium (- 4), ◗ ‑ detectable growth only on Low Stringency medium (- 3), 🞇 ‑ negative.

**Supplemental Table 4.**

| **N** | **Protein 1** | **Protein 2** | **Method** | **Reference** | **Species** |
| --- | --- | --- | --- | --- | --- |
| 1 | EMD | LZIP | Two-hybrid  Two-hybrid | **Rual et al, 2005**  **Rolland et al, 2014** | *H. sapiens*  *H. sapiens* |
| 2 |  |  |  |  |  |
| 3 | EMD | SYNE1 | Reconstituted Complex | **Mislow et al, 2002** | *H. sapiens* |
| 4 | EMD | LMNA | Affinity Capture-MS | **Holaska et al, 2007** | *H. sapiens* |
| 5 | LAP1 | COPS5 | Affinity Capture-MS | **Bennett et al, 2010; Matsumura et al, 1992** | *H. sapiens* |
| 6 | TGFB1 | SMAD2,3 | Co-localization | **Kahata et al, 2004** | *H. sapiens* |
| 7 | LAP1 | [LMNA](http://thebiogrid.org/201176/summary/mus-musculus/lmna.html) | Affinity Capture-MS | **Kubben et al, 2005** | *M. musculus* |
| 8 | LMNA | SYNE1 | Affinity Capture-Western, Reconstituted Complex | **Mislow et al, 2002** | *H. sapiens* |
| 9 | SYNE1 | MUSK | Affinity Capture-Western Two-hybrid | **Apel et el, 2000**  **Rolland et al, 2014** | *H.sapiens*  *H.sapiens* |
| 10 | MUSK | NEDD8 | Affinity Capture-Western | Del Rincón et al, 2010 | *H.sapiens* |
| 11 | NEDD8 | COPS6, COPS5 | Reconstituted Complex | **Birol et al, 2014**  **Jones et al, 2008** | *H.sapiens* |
| 12 | NEDD8 | SMAD3  SMAD4 | Affinity Capture-MS | **Bonacci et al, 2014** | *H.sapiens* |
| 13 | SMAD3 | SKI | Reconstituted Complex | **Chen et al, 2007** | *H.sapiens* |
| 14 | SMAD3 | Myod1 | Two-hybrid | **Ravasi et al, 2010** | *M. musuclus* |
| 15 | SMAD4 | Cops5 | Reconstituted Complex | **Sangadala et al, 2014** | *H. sapiens* |
| 16 | SKI | SKIP | Two-hybrid  Reconstituted Complex  Affinity Capture-Western | **Prathapam et al, 2001**  **Dahl et al, 1998**  **Tabata et al, 2009** | *H. sapiens* |
| 17 | HMOX1 | LZIP | Two-hybrid  Two-hybrid | Yu et al, 2011  **Rolland et al, 2014** | *H. sapiens*  *H. sapiens* |
| 18 | COPS5 | LZIP | Affinity Capture-Western  Co-localization  Two-hybrid  Reconstituted Complex | DenBoe et al, 2013 | *H. sapiens* |
| 19 | SKIP | Myod1 | Affinity Capture-Western | **Kim et al, 2011** | *H. sapiens* |
| 20 | HMOX2 | COPS6 | Two hybrid | **Stelzl et al, 2005** | *H.sapiens* |
| 21 | DMD | DAG1 | Affinity Capture-Western | **Jung et al, 1995** | *H.sapiens* |
| 22 | DMD | SGCB | Affinity Capture-MS | **Johnson et al, 2012** | *M. musuclus* |
| 23 | DMD | SGCZ | Affinity Capture-Western | **Wheeler et al, 2002** | *M. musuclus* |
| 24 | SGCB | SGCZ | Affinity Capture-Western | **Wheeler et al, 2002** | *M. musuclus* |
| 25 | DAG1 | SGCA | Affinity Capture-Western | **Matsumura et al, 1992** | *M. musuclus* |
| 26 | FLNC | SGCG  SGCD | Two-hybrid  Reconstituted complex | Thompson et al, 2000 | *H. sapiens* |
| 27 | SGCG | EP300 | Reconstituted complex | **Fryer et al, 2004** | *H. sapiens* |
| 28 | EP300 | SMAD3 | Two-hybrid  Affinity Capture-Western | Nishihara et al, 1999 | *H. sapiens* |
| 29 | EP300 | MYOD1 | Affinity Capture-Western  Reconstituted complex | **Yuan et al, 1996** | *H. sapiens* |
| 30 | SKIP | PABP2 | Two-hybrid  Affinity Capture-Western | **Kim et al, 2001** | *M. musuclus*  *H. sapiens* |
| 31 | SKIP | VDR | Far western  Affinity Capture-Western  Reconstituted complex | **Baudino et al, 1998** | *H. sapiens* |
| 32 | SKIP | SMAD2,3,4 | Two-hybrid  Reconstituted complex | **Leong et al, 2001** | *H. sapiens* |
| 33 | NEDD8 | COPS5 | Affinity Capture-MS | **Hillery et al, 1991** | *H. sapiens* |
| 34 | SGCB | SGCD  SGCG | Cross-linking | **Chan et al, 1998** | *M. musuclus* |
| 35 | SGCG | SGCD |  | **Chan et al, 1998** | *M. musuclus* |
| 36 | SGCA | SGCB | Affinity Capture-Western | **Matsumura et al, 1992** | *M. musuclus* |
| 37 | SGCA | SGCD | Affinity Capture-Western | **Matsumura et al, 1992** | *M. musuclus* |
| 38 | LAP1 | EMD | Affinity Capture-Western | **Shin et al, 2013** | *H. sapiens* |

The binary interactions were found in databases IntAct, BioGrid and String followed by manual curation.

**
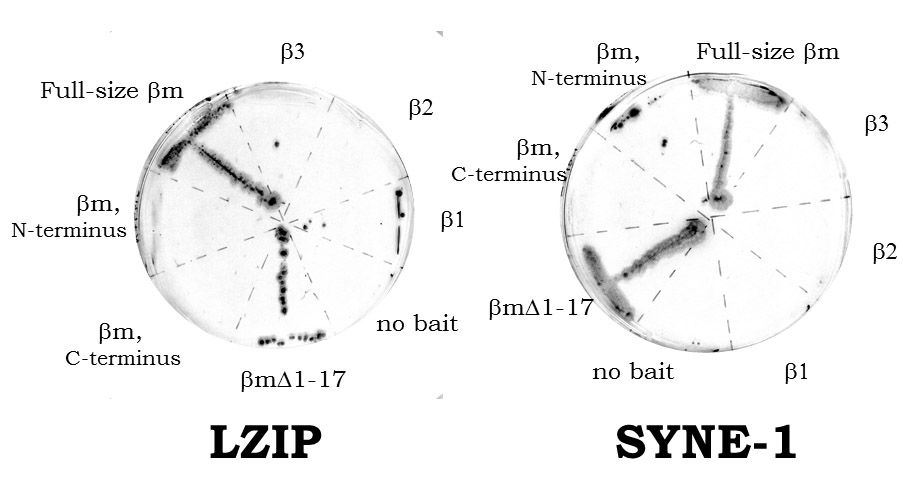
**

**Supplemental Figure 1. Typical results of yeast two-hybrid assays of specificty of interactions between BetaM and identified proteins.** Left panel – interaction with CREB3/LZIP, right panel – interactions with Syne-1.

**ex1 ex2**

**Hum MRRQLRSRRAPSFPYSYRYRLDDPDEANQNYLADEEEEAEEEARVTVVPKSEEEEEEEEK 60
Mus MRRQLRSRRAPAFPYGYRYRLDDQDEANHNYLADEEEEAEEEAQVMMVPGLEEEEEEEEG 60
Opo MERNSKSAEAEDISLNISSKPENENEGNQLHSEDRDEDEAQA------------------ 42**

**Pla MEKNPRAQEAESLPQSFPLKLENQDEGNQRLSEDREGETSETA----------------- 43**

**Chi *MEPGMEMNTASEGGTRRGP--ENKHEEKVQDPNRGEAETKAE------------------ 40
Xen MATTAGEQANYLQSA------DSMSDGRQHHPEEAGEKKQEE------------------ 36
Puf MEPAEGGVEEKPLKSI-----PHKVILKHGQELEEEQEELAEHQPLEQEDLNFE------ 49**

**ex2 ex3
Hum EEEEEEEKEEEEGQGQPTGNAWWQKLQIMSEYLWDPERRMFLARTGQSWSLILLIYFFFY 120
Mus KEEEEE-REEEEGQGQSTGSAWWRKLQIVNEYLWDPEKRMSLARTGQSRSLILVIYFFFY 119
Opo ----------------TEKWSLAAKMQKVKNYLWDPEKREFLGRSGQSWSLILLFYLIFY 86**

**Pla ------------------RRSWDDIMQQVKTYLWDPEKREFLGRSGQSWGLILFFYFILY 85**

**Chi ----------------MGNKTWADLAGEMKTFLWNPEERTCMGRTAKSWGLILLFYFIFY 84
Xen -----------------QKKSWGEWLQDLKIFIWNPEKKEVLGRDKKSWALILLFYFILY 79
Puf ----------RWKRRPLPKRTLHQKIDDLKTYLWNAETNEFMGRSGKSWSLILLFYAALY 99
 =membrane=**

**Supplemental Fig. 2. Multiple alignment of N-terminal cytodomains of homologous vertebrate BetaM proteins.** Hum – *Homo sapiens*, Mus – *Mus musculus*, Opo - *Monodelphis domestica*, Pla - *Ornithorhynchus anatinus*, Chi – *Gallus gallus*, Xen – *Xenopus laevis*, Puf – *Tetraodon nigroviridis*.


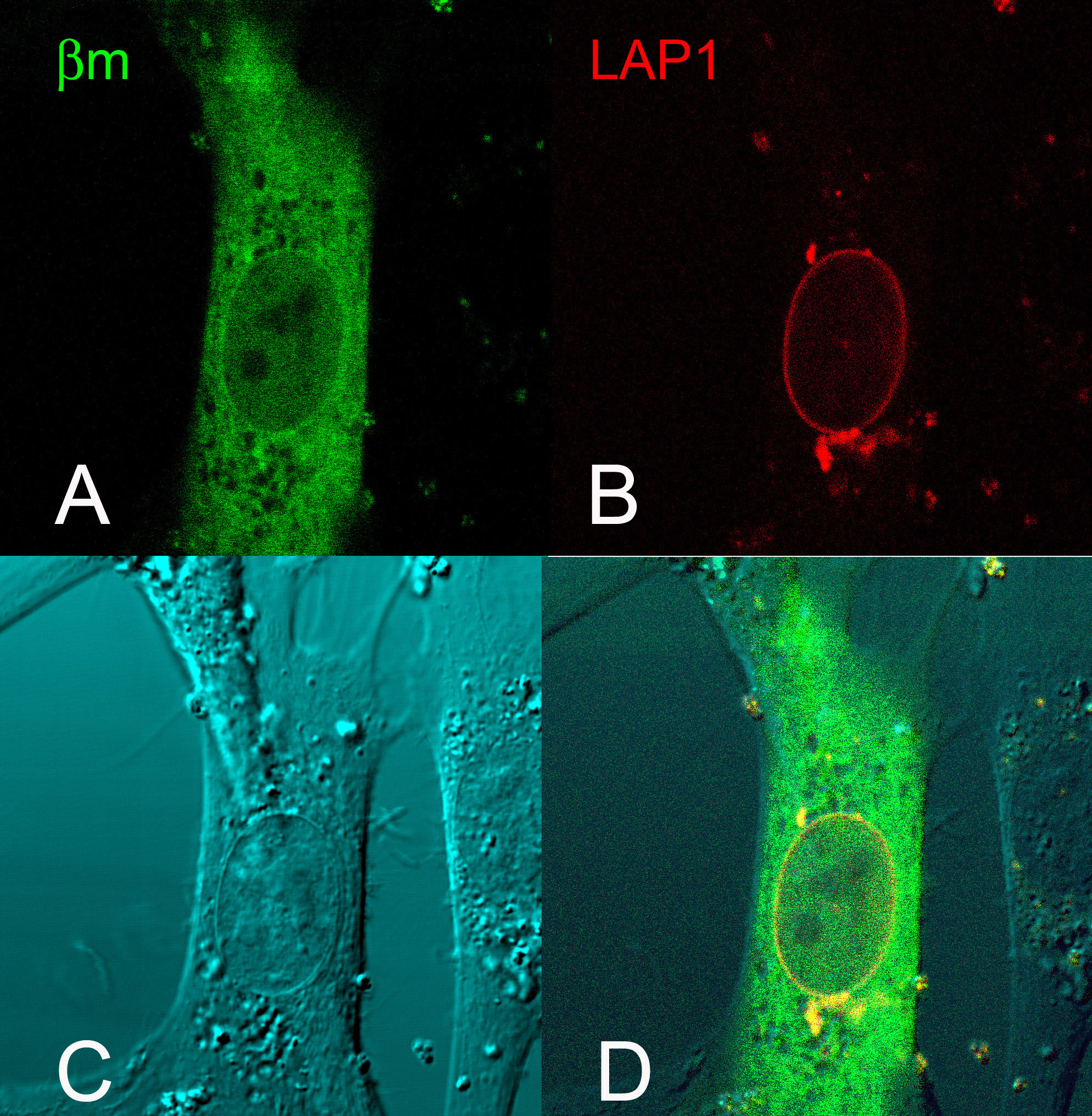


**Supplemental Figure 3.** Confocal imaging of subcellular distribution of βm and LAP1 tagged with fluorescent proteins in living cells. Mouse C2C12 myoblasts were transfected with plasmids encoding the BetaM-GFP and LAP1-RFP chimera. Green fluorescence of βm tagged with green fluorescent protein (A), red fluorescence of LAP1 tagged with red fluorescent protein (B). (C) Phase contrast image of the cells. (D) A, B, and C images merged.

**Supplemental references**

Apel, E.D., Lewis, R.M., Grady, R.M., Sanes, J.R. Syne-1, a dystrophin- and Klarsicht-related protein associated with synaptic nuclei at the neuromuscular junction. *J Biol Chem* **275**, 31986-31995 (2000)

Baudino, T.A. *et al.* Isolation and characterization of a novel coactivator protein, NCoA-62, involved in vitamin D-mediated transcription. *J Biol Chem*; **273**, 16434-16441 (1998)

Bennett, E.J., Rush, J., Gygi, S.P., Harper, J.W. Dynamics of cullin-RING ubiquitin ligase network revealed by systematic quantitative proteomics. *Cell* **143,** 951-965 (2010)

Birol, M. *et al.*. Structural and biochemical characterization of the Cop9 signalosome CSN5/CSN6 heterodimer. *PLoS One* **9**, e105688 (2014)

Bonacci, T. *et al*. Identification of new mechanisms of cellular response to chemotherapy by tracking changes in post-translational modifications by ubiquitin and ubiquitin-like proteins. *J Proteome Res* **13**, 2478-2494 (2014)

Chan, Y.M., Bönnemann, C.G., Lidov, H.G., Kunkel, L.M. Molecular organization of sarcoglycan complex in mouse myotubes in culture. *J Cell Biol* **143**, 2033-2044 (1998)

Chen, W. *et al.* Competition between Ski and CREB-binding protein for binding to Smad proteins in transforming growth factor-β signaling. *J Biol Chem* **282**, 11365-11376 (2007)

Dahl, R., Wani, B., Hayman, M.J. The Ski oncoprotein interacts with Skip, the human homolog of Drosophila Bx42. *Oncogene* **16,** 1579-1586 (1998)

Del Rincón, S.V. *et al*. Development and validation of a method for profiling post-translational modification activities using protein microarrays. *PLoS One* **5,** e11332 (2010)

DenBoe, L.M. *et al*. JAB1/CSN5 inhibits the activity of Luman/CREB3 by promoting its degradation. *Biochim Biophys Acta* **1829**, 921-929 (2013)

Fryer, C.J., White, J.B., Jones, K.A. Mastermind recruits CycC:CDK8 to phosphorylate the Notch ICD and coordinate activation with turnover. *Mol Cell* **16,** 509-520 (2004)

Hillery, C.A., Smyth, S.S., Parise, L.V. Phosphorylation of human platelet glycoprotein IIIa (GPIIIa). Dissociation from fibrinogen receptor activation and phosphorylation of GPIIIa in vitro. *J Biol Chem* **266,** 14663-14669 (1991)

Holaska, J.M., Wilson, K.L. An emerin "proteome": purification of distinct emerin-containing complexes from HeLa cells suggests molecular basis for diverse roles including gene regulation, mRNA splicing, signaling, mechanosensing, and nuclear architecture. *Biochemistry* **46,** 8897-908 (2007)

Jones, J. *et al*. A targeted proteomic analysis of the ubiquitin-like modifier nedd8 and associated proteins. *J Proteome Res* **17,** 1274-1287 (2008)

**Johnson, E.K. *et al.* Proteomic analysis reveals new cardiac-specific dystrophin-associated proteins. *PLoS One* 7, e43515 (2012)**

**Jung, D. *et al*. Identification and characterization of the dystrophin anchoring site on β-dystroglycan. *J Biol Chem*; 270, 27305-27310 (1995)**

Kahata, K. *et al*. Regulation of transforming growth factor-β and bone morphogenetic protein signalling by transcriptional coactivator GCN5. *Genes Cells* **9,** 143-151 (2004)

Kim, Y.J. *et al*. The product of an oculopharyngeal muscular dystrophy gene, poly(A)-binding protein 2, interacts with SKIP and stimulates muscle-specific gene expression. *Hum Mol Genet* **10,** 1129-1139 (2001)

Kotorashvili, A. *et al*. Anterograde transport of surfactant protein C proprotein to distal processing compartments requires PPDY-mediated association with Nedd4 ubiquitin ligases. *J Biol Chem* **284,** 16667-16678 (2009)

Kubben, N. *et al*. Identification of differential protein interactors of lamin A and progerin. *Nucleus* **1,** 513-525 (2010)

**Leong, G.M. *et al.* Ski-interacting protein interacts with Smad proteins to augment transforming growth factor-β-dependent transcription. *J Biol Chem* 276, 18243-18248 (2001)**

**Matsumura, K. *et al.* Association of dystrophin-related protein with dystrophin-associated proteins in mdx mouse muscle. *Nature* 360, 588-591 (1992)**

Mislow, J.M. *et al.* Nesprin-1α self-associates and binds directly to emerin and lamin A in vitro. *FEBS Lett* **525,** 135-140 (2002)

**Nishihara A., *et al.* E1A inhibits transforming growth factor-beta signaling through binding to Smad proteins. *J Biol Chem* 274, 28716-28723 (1999).**

Prathapam, T., Kühne, C., Hayman, M., Banks, L. Ski interacts with the evolutionarily conserved SNW domain of Skip. *Nucleic Acids Res* **29**, 3469-3476 (2001)

Ravasi, T. *et al.* An atlas of combinatorial transcriptional regulation in mouse and man. *Cell* **140,** 744-752 (2010)

Rolland, T. *et al.* A proteome-scale map of the human interactome network. *Cell* **159,** 1212-1226 (2014)

Rual, J.F. *et al.* Towards a proteome-scale map of the human protein-protein interaction network. *Nature* **437***,* 1173-1178 (2005)

Sangadala, S. *et al*. Characterization of a unique motif in LIM mineralization protein-1 that interacts with jun activation-domain-binding protein 1. *Mol Cell Biochem* **385**, 145-157 (2014)

**Shin, J.Y. *et al*. Lamina-associated polypeptide-1 interacts with the muscular dystrophy protein emerin and is essential for skeletal muscle maintenance. *Dev Cell* 26, 591-603 (2013)**

Stelzl, U. *et al.* A human protein-protein interaction network: a resource for annotating the proteome. *Cell* **122,** 957-968 (2005)

Tabata, T., Kokura, K., Ten Dijke, P., Ishii, S. Ski co-repressor complexes maintain the basal repressed state of the TGF-β target gene, SMAD7, via HDAC3 and PRMT5. *Genes Cells* **14,** 17-28 (2009).

Thompson, T.G. *et al.* Filamin 2 (FLN2): A muscle-specific sarcoglycan interacting protein. *J Cell Biol* **148,** 115-126 (2000)

**Wheeler, M.T., Zarnegar, S., McNally, E.M. ζ-sarcoglycan, a novel component of the sarcoglycan complex, is reduced in muscular dystrophy. *Hum Mol Genet* 11, 2147-2154 (2002)**

Yu, H. *et al.* Next-generation sequencing to generate interactome datasets. *Nat Methods* **8**, 478-480 (2011)

Yuan, W. *et al*. Human p300 protein is a coactivator for the transcription factor MyoD. *J Biol Chem*; **271,** 9009-9013 (1996)
